# Applying Raman Spectroscopy for Real-Time Monitoring of Ultra- and Diafiltration Steps in Viral Vector Purification

**DOI:** 10.3390/pharmaceutics18081016

**Published:** 2026-08-17

**Authors:** Cláudia S. Paiva, Hadi El Radi, Diogo Chagas, Kévin Grollier, Johan Cailletaud, Sébastien Delacroix, Paolo Gabaldi, Tiago Q. Faria, Cristina Peixoto

**Affiliations:** 1 iBET, Instituto de Biologia Experimental e Tecnológica, Apartado 12, 2781-901 Oeiras, Portugal; 2 ITQB NOVA, Instituto de Tecnologia Química e Biológica António Xavier, Universidade Nova de Lisboa, Av. da República, 2780-157 Oeiras, Portugal; 3 Merck Millipore SAS, 13 Chemin du Vieux Chêne, 38240 Meylan, France; 4 NOVA FCT, NOVA School of Science and Technology, Universidade Nova de Lisboa, 2829-516 Caparica, Portugal; 5 Merck Life Science, 39 Route Industrielle de la Hardt, 67129 Molsheim, France; 6 Merck Life Science, Via Monte Rosa, 93, 20149 Milan, Italy

**Keywords:** adeno-associated virus, chemometrics, critical quality attributes, downstream process, lentiviral vectors, process analytical technology, Raman spectroscopy

## Abstract

**Background:** Process analytical technology (PAT) enhances product quality by monitoring and controlling critical quality attributes (CQAs), offering better insights into process performance. Raman spectroscopy is a promising PAT tool to support the development of control systems for continuous and automated processes. In this study, Raman spectroscopy was used to monitor in real-time ultra- and diafiltration (UF/DF) of adeno-associated virus (AAV) and lentiviral vector (LV). **Method:** The incorporation of a Raman probe in a tangential flow filtration (TFF) system, when operated in open loop, enabled spectral acquisition during a five-fold concentration of clarified bulk followed by a buffer exchange to PBS with five diafiltration volumes. **Results:** Principal Component Analysis reduced the dimensionality of the training set, and a Partial Least Squares model was developed for each parameter in each phase. After validation, Raman monitoring platforms showed good performance in predicting CQA of both viral vectors. Batch-to-batch variability was identified as the principal source of spectral variation, and its impact on UF/DF operation was observed. **Discussion:** This study emphasises the potential of Raman spectroscopy as a PAT for monitoring and control strategies in the downstream processing of viral vectors. **Conclusions:** These monitoring platforms enhance understanding of TFF’s operation and enable timely decision making by providing real-time information on multiple parameters.

## 1. Introduction

The development of monitoring platforms for bioprocesses has been improving process efficiency and product quality. Implementing precisely controlled operations minimises performance deviation and errors by overtaking the conventional methodology of relying solely on end-of-batch quality control testing [1,2]. Early identification of outliers allows to reduce resource consumption, avoid unnecessary costs and enhance productivity. A cornerstone for the transition to autonomous manufacturing is adopting process analytical technology (PAT). PAT systems are designed to analyse and control processes by real-time measurement of critical process parameters (CPPs) and/or critical quality attributes (CQAs) of products or related materials. This framework supports the application of quality by design (QbD) in manufacturing, supervising risks and predicting outcomes throughout operations, according to the historical process knowledge and model-based approach [3,4,5].

As batch-to-batch variability may cause product failure, regulatory issues and potential risks to patients, strategies to minimise batch inconsistency are essential [6]. Since 2004, the US Food and Drug Administration (FDA) and European Medicines Agency (EMA) have nurtured the use of PAT framework and QbD in earlier pharmaceutical development phases. Within the PAT technologies used in the pharmaceutical industry, spectroscopic sensors are preferred for allowing (1) in-line measurements of bioprocess parameters without compromising sterility, (2) non-invasive and non-destructive information, (3) short response time and (4) simultaneous evaluation of multiple variables (physical, chemical and biological properties of product and/or relatives) [7,8,9]. Studies exploring Raman spectroscopy as a PAT for quality control testing of compositional and chemical characterisation of active pharmaceutical ingredients (APIs), crystallisation state, excipient and impurity detection [10,11,12,13,14] or for process monitoring [15,16,17,18,19,20,21] have been increasing significantly, reflecting the continued interest in applying this technique in the pharmaceutical industry.

Raman spectroscopy is a vibrational technique based on light-scattering effects. When a visible or near-infrared monochromatic laser interacts with a molecule, it polarises the molecular electron cloud, resulting in scattered photons with higher (anti-Stokes Raman scattering) or lower (Stokes Raman scattering) energy compared with the excitation radiation. The Raman scattering has low intensity and occurrence. Customisation of laser power and excitation time offers valuable information about chemical composition and molecular structure. Its specificity acts as a high-resolution fingerprint for identifying and quantifying molecules, ideal for monitoring purposes. Each Raman shift in the spectra represents a vibrational energy of a single chemical bond, whilst the intensity reflects the polarizability and abundance, and the peak width provides structural information [9,22]. Accordingly, it monitors each CQA individually by selecting the spectral zone where its chemical bonds are represented, instead of using the total signal. As a soft sensor, it relies on data-driven and model-driven principles to indirectly measure, quantify and correlate easy-to-measure variables and difficult-to-measure parameters [23,24]. Data quality plays a critical role in model performance and restricts its use to the process phase for which it was developed. A model is weakly transmissible across processes and facilities without adaptation. Digital twins combined with transfer learning algorithms have been demonstrated as a potential strategy for transferring models between operating environments and establishing control strategies to reduce process variability [25,26,27].

In bioprocessing, Raman spectroscopy is frequently applied in the upstream to accurately predict the concentration of metabolites, amino acids [28], product of interest, viable cell density and total cell density [29,30,31,32,33]. The spectral analysis focuses on the fingerprint region of proteins between 600 cm^−1^ and 1800 cm^−1^, where carbonyl (C=O) and amine (N-H) groups of the protein backbone are predominant. Within this region, hydrogen bonding highlights α-helices, β-sheets and/or random coils representative of the secondary structure [34]. The tertiary structure is deduced from disulfide bonds and aromatic amino acids of side chains inserted in 500–550 cm^−1^ Raman shift [35]. Li et al. (2018) [36] showed the efficiency of Raman spectroscopy in monitoring the glycosylation profile of monoclonal antibodies (mAbs) in CHO cell cultures, emphasising its ability to distinguish extremely similar molecules in complex mixtures. Differences in mAbs’ tertiary structure promoted by side-chain bonds induce variability in molecular polarisation and in Raman activity. For quality control, Moor et al. (2018) [37] exploited Raman spectroscopy to identify early-stage viral infection in human cells, and Morder et al. (2022) [38] established a method to determine lentiviral vector infectious titer without cellular infection. Advances in optical components like high-power lasers and fibre-optical probes are reducing the acquisition time to seconds, allowing faster measurements. The technology, combined with the extended knowledge of Raman’s capabilities, allows its use to expand into downstream processing. Although, the dynamic nature of downstream process is a challenge due to (1) the impact of operation speed on cycle and acquisition time, (2) the impact of concentration range on dynamics during operation, (3) the environmental impact (solvents, temperature, pressure, etc.) on Raman’s sensitivity, (4) the limited place to implement the probe and (5) the cost-effective benefit of using Raman spectroscopy compared with other acquisition methods [4,39]. Feidl et al. (2019) [40] successfully used Raman spectroscopy to monitor the breakthrough of mAbs in a Protein A column by implementing a multi-pass flow cell between the elution stream and the sample fractionator of the chromatography system. Alternatively, Rolinger et al. (2023) [41] showed the power of Raman spectroscopy combined with an extended Kalman filter to monitor ultra- and diafiltration (UF/DF) operation of bispecific monoclonal antibodies (sbAbs) by immersing the probe into the retentate reservoir. Recently, Heyer-Müller et al. (2025) [42] exploited the accuracy of a partial least squares (PLS) model to measure phosphate- and citrate-buffered lysozyme mixtures in chromatography-based purification into a UF/DF operation for predicting concentration trends. The compromise between operation speed and acquisition time makes UF/DF a suitable downstream operation to implement Raman spectroscopy. Few applications of Raman spectroscopy in downstream processing are known; nevertheless, they provide significant benefits in showcasing Raman spectroscopy’s robustness and consistency for measuring product concentration, purity and excipient concentration throughout the unit operations.

In this work, the use of Raman spectroscopy for process monitoring was extended to the downstream process of viral vectors, establishing a real-time monitoring platform for UF/DF of adeno-associated virus (AAV) and lentiviral vector (LV). An initial technology application shows the characterisation of AAV and LV spectra, highlighting intrinsic differences previously undetectable by reference analytical methods. The correlation between Raman spectra and CQAs enabled the simultaneous measurement of multiple parameters. The training set allowed the development of models for UF spotlighting viral vector retention and models for DF following *ds*DNA and protein clearance. The validation set enabled verification of the model’s predictive performance. The in-line Raman measurements offer direct understanding of UF/DF of viral vectors, advance operation efficiency, and minimise sampling demands and quality control analyses throughout operation. These results demonstrate the potential of Raman spectroscopy as a promising PAT for bioprocessing engineering in gene therapy.

## 2. Materials and Methods

### 2.1. Clarified Viral Vector Material

HEK293T cells (ATCC^®^ CRL/3216TM) from the American Type Culture Collection (ATCC, Manassas, VA, USA) were adapted to suspension and cultivated in BalanCD^®^ HEK293 medium (FUJIFILM IrvineScientific, Santa Ana, CA, USA) supplemented with 4 mM GlutaMAX^TM^ (ThermoFisher Scientific^TM^, Carlsbad, CA, USA) at 37 °C and 90 rpm (INFORS HT, Bottmingen, Switzerland) with a humidified atmosphere of 8% CO_2_ in air. Cells were maintained until 4.0 × 10^6^ cells/mL and were routinely passed. Cell density and viability were measured using the image-based cell analyser Vi-CELL BLU Cell Viability Analyser (Beckman Coulter, Miami, FL, USA) following the trypan blue exclusion method.

LV-GFP was expressed transiently using the fourth-generation *p*ALD-Lenti System (Aldevron, Fargo, ND, USA) and PEIpro^®^ Transfection Reagent (Polyplus Transfection^®^, Illkirch, France) at a mass ratio of 1:2 (DNA:PEI). Cells were transfected at 2.5 × 10^6^ cells/mL in a 2 L shake flask (500 mL working volume), considering 1 µg of total DNA/1.0 × 10^6^ cells. At 48 h post-transfection, VSV-G pseudotyped LVs were harvested when cell density was around 5.0 × 10^6^ cells/mL and treated with Benzonase^®^ endonuclease (Merck KGaA, Darmstadt, Germany) at 100 U/mL and 2 mM MgCl_2_. The DNA digestion was performed at 37 °C for 30 min. The harvest was clarified using a 3-stage cascade filtration set: (1) Sartopure^®^ PP3 Midicaps^®^, 20 µm pore size (Sartorius, Göttingen, Germany); (2) Sartoclean^®^ CA Midicaps^®^, 3 + 0.8 µm pore size (Sartorius, Germany); and (3) Sartopore^®^ 2 300, 0.80 + 0.45 µm pore size (Sartorius, Germany). The clarified LV material had a turbidity inferior to 10 FNU.

AAV serotype 8 (AAV8)-GFP was expressed transiently using the *p*AAV System (PlasmidFactory, Bielefeld, Germany) and PEIpro^®^ Transfection Reagent (Polyplus Transfection^®^, Illkirch, France) at a mass ratio of 1:1.5 (DNA:PEI). Cells were transfected at 2.0 × 10^6^ cells/mL in a 2 L shake flask (500 mL working volume), considering 1.5 µg of total DNA/1.0 × 10^6^ cells. At 72 h post-transfection, AAV8 were harvested when cell density was around 4.0 × 10^6^ cells/mL and viability was ~70%. Cells were lysed with 50 mM Tris, 1% (*v*/*v*) Tween 20 and 2 mM MgCl_2_ at pH 8.0, with addition of 50 U/mL of Benzonase^®^ endonuclease (Merck KGaA, Germany). The lysis and nuclease treatment were performed at 37 °C for 2 h with subsequent addition of 500 mM NaCl to stabilise AAV8. The harvest was clarified by centrifugation at 2246 *g* (Avanti^®^ J-HC, Beckam Coulter Life Sciences, Brea, CA, USA) for 10 min at 20 °C and filtered using a Nalgene^TM^ Rapid-Flow^TM^ Sterile Single-Use Vacuum Filter Unit (ThermoFischer Scientific^TM^, USA) with a 0.22 µm pore size polyethersulfone membrane. The clarified AAV8 material used for the training set had a turbidity inferior to 10 FNU, and for the validation set, a turbidity lower than 25 FNU.

### 2.2. Ultra- and Diafiltration Operation

**Figure 1:**
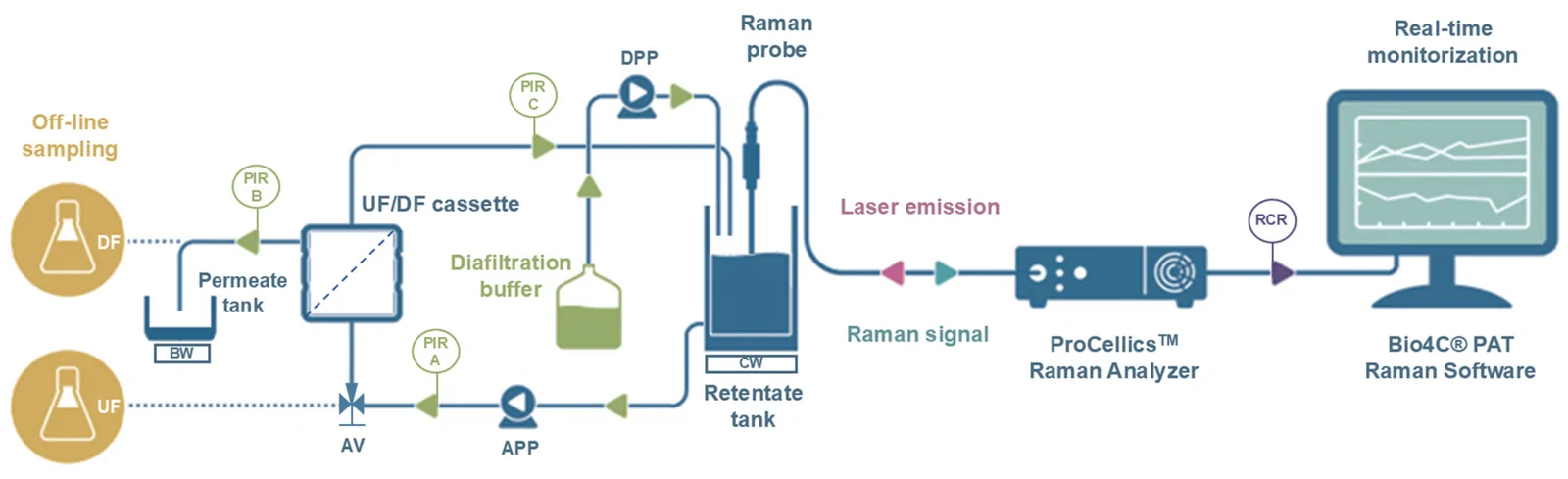
Instrumentation diagram of TFF setup with the Raman probe inserted into reservoir vessel. Pressure sensors and balances are critical elements of a process control system. Data were captured both automatically and manually. Sampling in UF was performed using a three-way valve from the feed stream and in DF from the permeate stream. The change from the UF to DF step is associated with the start of diafiltration buffer pumping. The Figure was adapted from Merck Millipore website. A: feed; APP: peristaltic feeding pump; AV: feed valve; B: permeate; BW: permeate weight; C: retentate; CW: retentate weight; DF: diafiltration; DPP: peristaltic diafiltration buffer pump; UF: ultrafiltration; PIR: pressure indicating and recording; RCR: Raman communication and recording.

In this study, a 100 kDa Biomax Pellicon^®^ XL50 cassette (Merck KGaA, Germany) and a 300 kDa Ultracel Pellicon^®^ XL50 cassette (Merck KGaA, Germany) with a filtration area of 50 cm^2^ were used to UF/DF AAV8 and LV, respectively. The UF/DF operation was carried out in Cogent^®^ Lab 150 Systems (Merck KGaA, Germany) for the training set and in a TFF-assembled system monitored through the SciLog pressure system for the validation set (Figure 1). No differences were observed in Raman spectra between batches when using both systems. The cassettes were operated at a constant transmembrane pressure (TMP) of 0.50 bar, restricting the operation procedure to a maximum TMP of 1.0 bar and a maximum feed pressure of 1.5 bar. Operational feed flux was established at 300 LHM, and permeate flux was kept constant at 24 LHM by manually adjusting the opening and closing of the retentate line. Cassettes were sanitised according to the manufacturer’s instructions with 0.1 M NaOH at 100 LHM and 100 L/m^2^. Prior to UF/DF operation, the systems were washed with water until the conductivity and pH were inferior to 5 mS/cm and 7, respectively. The Raman probe was submerged in the biological material in the reservoir vessel during UF/DF.

Seven independent batches were used as a training set for model development. The biological material was initially recirculated for 10 min to 15 min. In the UF phase, the sampling was performed from the cassette inlet. After reaching a 5-fold concentration, an external peristaltic pump was connected to the reservoir vessel, and PBS (Merck KGaA, Germany) was added continuously at 24 LHM as a diafiltration buffer. The external pump flux was adjusted to the permeate flux. In DF, sampling was performed from the permeate line. Permeate conductivity and pH were monitored during DF. After 5 diafiltration volumes, the external peristaltic pump stopped, and diafiltrate was recirculated counterclockwise for 5 min before sample collection. The system was flushed with PBS using 2 hold-up volumes. During operation, 500 µL samples from clarified bulk, each volume concentration factor, permeate, each diafiltration volume and the final diafiltrated product were collected and aliquoted for off-line measurement.

### 2.3. Raman Acquisition

The ProCellics™ Raman Analyser (Merck KGaA, Germany) instrument was operated in a single-channel configuration. The system is equipped with a 785 nm laser. Spectral data were collected between −80 cm^−1^ and 4000 cm^−1^ with a mathematical resolution of 3 cm^−1^. The system is equipped with a back-thinned charge-coupled device as a detector. At-line measurement of 1 mL sample of purified AAV and LV was performed in the probe compartment for spectral collection. On the contrary, in in-line measurement, the probe was immersed in the TFF reservoir vessel to collect the sample signal from clarified bulk during UF/DF operation. Aluminium foil was used to cover the reservoir vessel, ensuring no stray light would disturb the measurement. Laser excitation, spectral data collection and operation of the hardware were performed through the Bio4C^®^ PAT Raman Software (version 6.1, Merck KGaA, Germany) using an Ethernet connection between the hardware and a computer.

The Raman acquisition parameters used for LV were an integration time of 35 s with an average of 5 spectra, requiring 175 s per measurement, while for AAV8, an integration time of 8 s with an average of 5 spectra was used, requiring 40 s per measurement. A total of 7 batches were collected for the training set, and 1 batch was used for the validation set. More than 150 spectra were collected for each UF/DF run. In every run, 7 samples were collected during UF and 6 samples during DF at the same process stage. For each viral vector, 1050 spectra were collected, treated and processed per CQA for modelling. A total of 49 and 42 samples were characterised off-line using reference analytical methods to correlate with Raman spectra for UF and DF, respectively. LV batches were generated from biological quintuplicates sequentially produced, harvested, and clarified; the AAV8 batches were derived from biological triplicates. A new cassette was used in each UF/DF run to capture differences in performance within the collected Raman spectra.

### 2.4. Spectral Data Pre-Processing for Model Building

The averages of the Raman spectra collected during UF/DF operation were pre-processed between 350 cm^−1^ and 1775 cm^−1^, and 2800 cm^−1^ and 3000 cm^−1^, for all models using Standard Normal Variate (SNV) normalisation calculated in the water band region (Raman shift between 3100 cm^−1^ and 3700 cm^−1^). As the water band region in Raman spectra is assumed to be constant, it served as an internal standard for data treatment [43]. Accordingly, the entire spectrum was normalised to reduce inter-batch variability and variations in optical scattering [4]. Savitzky–Golay’s first derivative transformation [44] was applied to the normalised dataset using a second-order polynomial and a 5-point fitting window to decrease fluorescence and emphasise subtle spectral variations. A smoothing window size was defined as a trade-off between effective noise smoothing and spectral resolution. Spectra were visually inspected to identify and eliminate any potential atypical acquisitions. Variable selection was performed independently for each parameter through iterative testing of different variable subsets and evaluation of their respective model performances. All spectral data processing was performed within the Bio4C^®^ PAT Chemometric Expert module (Merck KGaA, Germany).

#### 2.4.1. Principal Component Analysis

The Principal Component Analysis (PCA) technique was applied to reduce the dimensionality of the pre-processed dataset [45]. The high-dimensional spectra data were transformed into a low-dimensional space. The technique preserves data variability by decomposing the initial data into new variables called principal components (PCs). The spectra intensity Matrix X (n × m) was generated, where n is the number of spectra, and m is the number of wavenumbers [46]. Consequently, PCs were computed sequentially. A subset of PCs was selected to explain more than 95% of the variance in the initial variables. Score plots were used to visualise sample clustering and outlier detection, while loading plots showed the spectral features of the observed variation. Patterns and sources of variability were identified in complex Raman spectral datasets. All PCA models and associated PCA plots were obtained with the Bio4C^®^ PAT Chemometric Expert module (Merck KGaA, Germany).

#### 2.4.2. Partial Least Squares

The correlation between Raman spectra and off-line values of CQAs and total protein for LV was performed using PLS regression. Latent variables were generated by the PLS algorithm using linear combinations of Raman shifts. Each latent variable maximises the variance in the Raman spectra, whilst optimising covariance between Raman spectra scores and those parameters [47]. The number of latent variables of each PLS model was optimised following a multi-criteria approach. The root-mean-square error of calibration (*RMSEC*) and the root-mean-square error of cross-validation (*RMSECV*) were minimised when the cumulative explained variance (*R*^2^*Y*) and the cumulative predicted variance (*Q*^2^*Y*) were maximised. All PLS models were calibrated within the Bio4C^®^ PAT Chemometric Expert module (Merck KGaA, Germany). The created model was applied to monitor UF/DF with a new and independent batch. To assess the model monitoring accuracy, the root-mean-square error of prediction (*RMSEP*), relative error (*RE*) and ratio of prediction to deviation (*RPD*) metrics were determined [48,49]. The *RMSE* formula (Equation (1)) was consistent for *RMSEC*, *RMSECV* and *RMSEP*, according to the dataset used in which the formula was applied. The *R*^2^*Y* represents the variance in the response variable *Y* (Equation (2)), and *Q*^2^*Y* indicates the predictive performance of the model through cross-validation (Equation (3)). The standardised *RE* (Equation (4)), expressed as a percentage, indicates the model accuracy across different units of measurement and depends on the parameter being modelled. The *RPD* (Equation (5)) is a performance metric used to evaluate the predictive consistency of a model, where *SD* is the standard deviation. A stable and accurate predictive model has an *RPD* higher than 2, whereas a poor predictive capacity has an *RPD* lower than 1.4 [49].

(1) $$RMSE=\sqrt{\frac{\sum_{i}^{n}{\left(y_{i}-{\hat{y}}_{i}\right)}^{2}}{n}},$$

(2) $$R^{2}Y=1-\frac{\sum_{i}^{n}{\left(y_{i}-{\hat{y}}_{i}\right)}^{2}}{\sum_{i}^{n}{\left(y_{i}-\bar{y_{i}}\right)}^{2}},$$

(3) $$Q^{2}Y=1-\frac{\sum_{i}^{n}{\left(y_{i}-{\hat{y}}_{i,CV}\right)}^{2}}{\sum_{i}^{n}{\left(y_{i}-\bar{y_{i}}\right)}^{2}},$$

(4) $$RE=\frac{RMSE}{y_{max}}\times 100,$$

(5) $$RPD=\frac{SD}{RMSEP},$$

These approaches evaluate the PLS model’s performance in predicting CQAs and total protein for LV from Raman spectra data and demonstrate the potential of Raman spectroscopy to monitor operation units in the downstream process of viral vectors.

### 2.5. Analytical Methods

#### 2.5.1. Total Particle Quantification

Total particle (TP) concentration in harvest, clarified, cassette retentate and final diafiltrate samples was assessed using ELISA Kits. For LV TP, the ELISA Kit Innotest^®^ HIV Antigen mAb (Fujirebio, Malvern, PA, USA) was used to quantify the amount of p24 protein. The samples were diluted according to the standard curve, which ranged from 150 to 25 pg p24/mL. The assay was performed following the manufacturer’s instructions. A ratio of 1.25 × 10^7^ TP/p24 ng was used to convert the amount of viral-coated protein to LV TP titer. The absorbance at 450 nm and 650 nm was measured on an Infinite^®^ M200 PRO NanoQuant (Tecan, Männedorf, Switzerland) microplate multimode reader. For AAV8, the AAV8 Titration ELISA Kit (Progen Biotechnik, Heidelberg, Germany) was used to measure the number of TP. The samples were diluted according to the standard curve, which ranged from 5.1 × 10^8^ to 8.0 × 10^6^ capsids/mL. The assay was performed according to the manufacturer’s instructions. Absorbance at 450 nm and 650 nm was measured on an Infinite^®^ M200 PRO NanoQuant (Tecan, Switzerland) microplate multimode reader, and the AAV8 titer was determined using 4PL regression.

#### 2.5.2. Viral Genome Extraction

The viral genome of AAV8 was quantified by *q*PCR. The *ss*DNA was isolated using the High Pure Viral Nucleic Acid Kit (Roche Life Science, Basel, Switzerland) according to the manufacturer’s instructions. For measuring full capsids, the probe (5′-TTGCCGTCCTCCTTGAAGTCGAT-3′) and eGFP-specific primers (forward primer, 5′-GAACCGCATCGAGCTGAA-3′ and reverse primer, 5′-TGCTTGTCGGCCATGATATAG-3′) were used. The quantification was performed in a LightCycler^®^ 480 (Roche, Switzerland) analyser with 40 cycles at 95 °C for 10 min, followed by 10 s at 62 °C.

#### 2.5.3. Transducing Particles Quantification

HEK293T cells (ATCC^®^ CRL/3216TM) from the American Type Culture Collection (ATCC, USA) were cultivated in Dulbecco’s modified Eagle’s medium (DMEM, ThermoFisher Scientific^TM^, USA) supplemented with 10% fetal bovine serum (ThermoFisher Scientific^TM^, USA) and 4 mM GlutaMAX^TM^ (ThermoFisher Scientific^TM^, USA) at 37 °C with a humidified atmosphere of 5% CO_2_. Cells were seeded in a 24-well plate at 0.6 × 10^5^ cells/cm^2^. After 24 h, the supernatant was removed, and the HEK293T cells were transduced with VSV-G-pseudotyped LV, assuming a multiplicity of infection ranging from 10^−1^ to 10^−4^. The viruses were serially diluted in fresh medium containing 8 µg/mL of Polybrene Transfection Reagent (Merck KGaA, USA) and 1% Penicillin-Streptomycin (Gibco^TM^, Neosho, MO, USA) before addition to cells. After 4 h post-transduction, 500 µL/well of fresh medium containing 1% penicillin–streptomycin (Gibco^TM^, USA) was added to the infected HEK293T cells. After 48 h post-transduction, the cells were trypsinised and resuspended in BD FACSFlow^TM^ Sheath Fluid (BD Biosciences, San Diego, CA, USA). Positive GFP cells, ranging from 5 to 20%, were measured by flow cytometry using FACSCelesta^TM^ (BD Biosciences, USA). The titer of VSV-G-pseudotyped LV-transducing units (TU/mL) was determined using Equation (6).

(6) $$Titer\;\left(TU/mL\right)=\frac{\%\;GFP\;positive\;cells÷100}{volume\;of\;transduction}\times viral\;dilution\;factor\times transduced\;cells,$$

#### 2.5.4. Total Protein and *ds*DNA Quantification

The total protein concentration was quantified using the Pierce^TM^ BCA Protein Assay Protein kit (ThermoFisher Scientific^TM^, USA) according to the manufacturer’s instructions. The standard calibration curve ranged from 2.0 to 0.02 mg/mL. The samples were serially diluted, and the absorbance at 562 nm was measured using an Infinite^®^ M200 PRO NanoQuant (Tecan, Switzerland) microplate multimode reader.

The *ds*DNA was assessed using the Quant-iT^TM^ Picogreen^®^ *ds*DNA assay kit (Invitrogen, USA) according to the manufacturer’s instructions. The standard calibration curve ranged from 2.0 to 0.02 µg/mL. The samples were serially diluted, and fluorescence was measured using an Infinite^®^ M200 PRO NanoQuant (Tecan, Switzerland) microplate multimode reader (excitation wavelength of 480 nm and emission wavelength of 520 nm).

#### 2.5.5. Turbidity Measurement

The turbidity of harvest, cell lysates and clarified samples was measured using a 2100 Qis Portable Turbidimeter (HACH, Loveland, CO, USA).

## 3. Results

### 3.1. Characterisation of Viral Vector Raman Spectra

**Figure 2:**
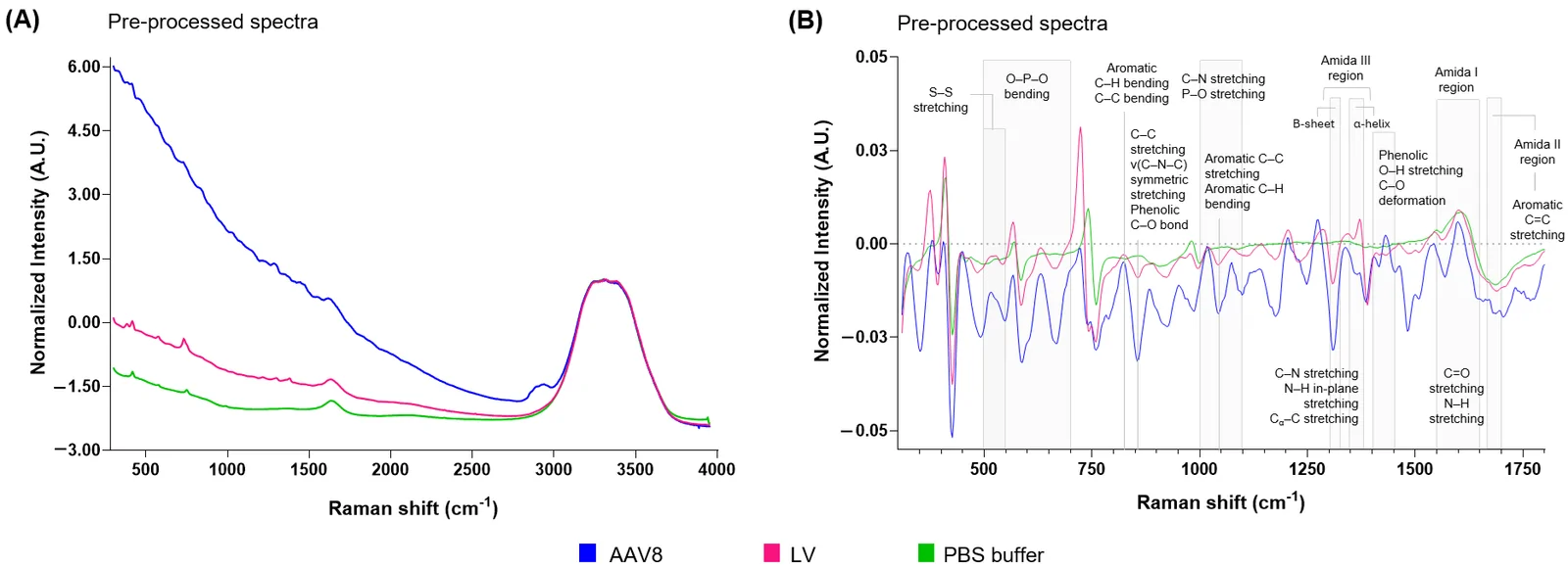
Pre-processed Raman spectra of AAV8 formulated in PBS (in blue), LV formulated in PBS (in magenta) and PBS buffer (in green) after (**A**) normalisation to the water band and (**B**) normalisation to the water band followed by derivatisation using a Savitzky–Golay filter, zoomed in on the 300–1750 cm^−1^ spectral region.

As an initial technology application, Raman spectroscopy was used at-line to measure purified samples of AAV8 and LV (Figure 2). Both viral vectors were formulated in PBS buffer, a low-frequency Raman-active buffer, to avoid buffer interference in the Raman spectra of the viral vectors. The total protein concentration remained comparable between samples (0.23 mg/mL for LV and 0.24 mg/mL for AAV8), with a high difference in residual *ds*DNA concentration (0.04 µg/mL for LV and 0.3 µg/mL for AAV8). Like proteins, the chemical bonds of *ds*DNA are expressed in the Raman spectra. However, DNA has a pronounced contribution between 2800 cm^−1^ and 3000 cm^−1^ Raman shifts. Similarly, both viral vectors have GFP as a reporter gene, and the transduced protein can increase the sample fluorescence. The high concentration of AAV8 titer compared with LV titer induces baseline differences ((6.58 ± 0.19) × 10^9^ TP/mL for LV; (1.55 ± 0.66) × 10^12^ TP/mL and (3.41 ± 2.2) × 10^11^ VG/mL for AAV). The spectra were analysed in the protein fingerprint region between 300 cm^−1^ and 1800 cm^−1^ to study spectral differences related to molecular structure and residue interactions. Spectral pre-processing includes normalisation to the water band (3100–3700 cm^−1^) and derivative treatment to minimise baseline shift and fluorescence artefacts, enabling, as much as possible, a non-biased spectral comparison without loss of compositional and structural information. Controlling potential sources of spectral variation minimises systematic errors during data collection.

Raman spectroscopy can distinguish the molecular fingerprints of AAV8 and LV. Each spectrum reflects a unique vibrational and structural composition (Figure 2A). The Raman signal of AAV8 exhibits a high baseline shift, despite the substantial reduction in acquisition time (8 s) compared with LV (35 s) due to the inherent fluorescence. A high Raman shift intensity suggests fundamental differences in the optical and molecular properties between viral vectors. Analysing the pre-processed spectra zoomed into the fingerprint region of 400 cm^−1^ to 1600 cm^−1^ reveals characteristic vibrational signatures consistent with proteins and DNA (Figure 2B). The amplitude of a Raman shift indicates the abundance of a chemical bond vibration in a molecule compared with the contribution of the formulation buffer. The significant difference in signals demonstrates that AAVs are more Raman-active than LV. Key spectral features at ~850 cm^−1^ are attributed to C-C stretching vibrations and ν(C-N-C) symmetric stretching of amino groups, confirming protein contributions in both vectors. Characteristic amide III bands around 1300 cm^−1^ to 1400 cm^−1^ are present in the spectra, as well as amide I bands in the 1550 cm^−1^ to 1650 cm^−1^ region showing amide I signatures of C=O stretching and N-H bending. The 1000 cm^−1^ to 1100 cm^−1^ region exhibits C-N stretching vibrations and phosphate group modes from residual nucleic acids. This observation is consistent with measured residual *ds*DNA. The lytic character of AAV8 promotes a high amount of free *ds*DNA impurity when compared with LV. The lysogenic character of LV results in lower impurity release. Furthermore, AAV8 displays more spectral features in regions associated with aromatic amino acids, particularly around 1050 cm^−1^, which can be attributed to phenylalanine ring breathing vibrations. The band at ~828 cm^−1^ observed in both viral vectors likely indicates tyrosine contributions, characteristic of para-substituted benzene ring breathing in tyrosine residues.

**Figure 3:**
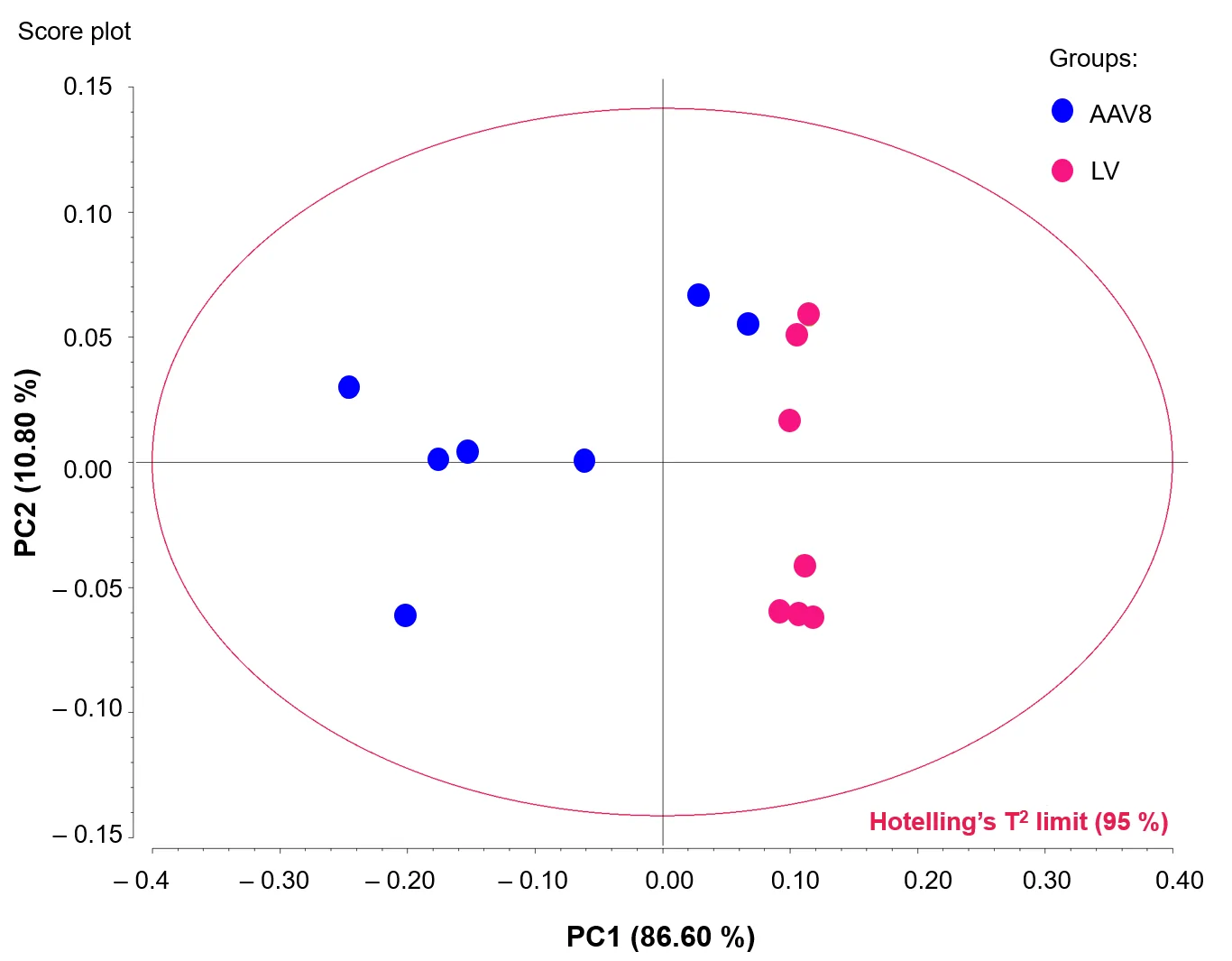
PCA score plot of Raman spectra collected from AAV8 (in blue) and LV (in magenta) pure samples. The first two principal components explain 97.40% of the total variance (PC1 = 86.60%; PC2 = 10.80%). The statistical analysis follows Hotelling’s T^2^ distribution within a 95% confidence interval.

Purified samples of formulated vectors in PBS were measured at-line and underwent a PCA analysis to assess spectral variability (Figure 3). The PCA score plot identified two sample groups, with PC1 (86.60%) and PC2 (10.80%) accounting for 97.40% of the total spectral variance. It showed that each viral vector has its characteristic features, where PC1 captures more variability in AAV samples than in LV samples. No outliers were detected in the data. Notably, AAV8 samples with low protein amounts appeared closer to the LV group without overlapping. In these cases, the spectral variance between AAV8 and LV was reduced, and the variance within intra-AAV8 samples increased. This suggests that the difference in protein content is the primary driver for the variability observed in the Raman fingerprint region.

### 3.2. Implementation of Raman Monitoring Platform in Ultra- and Diafiltration

The quantification of a parameter using Raman spectra is based on the correlation between off-line references and Raman shifts’ intensity for a group of chemical bond vibrations representative of each CQA. The model makes predictions associated with differences in all Raman shifts’ intensity and the concentration range of the training set. Fluorescence plays a critical role in quantification by masking the intensity of low Raman shifts and less representative chemical bonds through a baseline shift.

#### 3.2.1. Model Calibration for Adeno-Associated Virus Serotype 8

The Raman spectra of seven UF/DF runs were acquired with a frequency of 40 s per measurement to develop a chemometric model for each CQA of AAV8. During the model calibration, one batch was excluded from the UF dataset after being identified as an outlier by Hotelling’s T^2^ analysis. Compared with a purified sample, the Raman spectra of clarified material showed a high baseline shift due to the increase in fluorescence. This is derived from free protein content, viral vector titer and medium components. An optimal acquisition time is fundamental to attenuate measurement errors. In each run, samples were taken at specific time points to quantify the total protein, *ds*DNA, total particles and full capsids. The off-line references were matched up with the corresponding spectral measurement based on matching timestamps. The calibration data were leveraged to create four individual PLS regressions, each corresponding to one of the parameters to predict.

The calibration performance of PLS models was evaluated using k-fold cross-validation. It underscored the model’s efficiency in capturing analyte variability in the spectra throughout the UF/DF process steps (Table 1). In the UF phase, the total protein model exhibited an *R*^2^*Y* of 0.99 and a *Q*^2^*Y* of 0.97, indicating good predictive capability. This model used four latent variables ranging from 1.90 to 5.69 mg/mL. Similarly, the *ds*DNA model showed good performance, achieving an *R*^2^*Y* of 0.98 and a *Q*^2^*Y* of 0.94 using four latent variables. The *ds*DNA ranged from 3.57 to 30.39 µg/mL, and the model yielded a low *RMSECV* of 2.05. The total particle model achieved an *R*^2^*Y* of 0.93 and *Q*^2^*Y* of 0.82, with four latent variables ranging from 2.51 × 10^11^ to 4.09 × 10^12^ TP/mL. Although the *Q*^2^*Y* was slightly lower compared with the other models, it indicated good cross-validation performance. Therefore, the *RMSEC* value was 3.68 × 10^11^, and the *RMSECV* was 5.60 × 10^11^. For the full capsid model, the model displayed an *R*^2^*Y* of 0.98 and a *Q*^2^*Y* of 0.90 using five latent variables ranging from 1.35 × 10^11^ to 4.89 × 10^12^ VG/mL. The *RMSECV* of 1.97 × 10^11^ indicated a reasonable level of error. In the DF phase, the total protein model achieved an *R*^2^*Y* of 0.94 and a *Q*^2^*Y* of 0.67, employing five latent variables ranging from 0.00 to 1.15 mg/mL, with an *RMSECV* of 0.17. The *ds*DNA model recorded an *R*^2^*Y* of 0.87 and a *Q*^2^*Y* of 0.75, using three latent variables ranging from 0.30 to 4.00 µg/mL and an *RMSECV* of 0.37. The observed *Q*^2^*Y* of the total protein and *ds*DNA models for DF was lower than for the UF models. Relative to the low concentration, the limit of detection of Raman spectroscopy may have been reached. However, no overfitting was detected in the models. Overall, the PLS models demonstrated satisfactory performance across both UF and DF phases, affirming their reliability in quantifying AAV CQAs under evaluation.

#### 3.2.2. Model Calibration for Lentivirus Vector

The construction of chemometric models for LV involved gathering the Raman spectra from seven UF/DF runs, with spectra collected every 3 min. The time between measurements is more relevant in LV runs, requiring a longer integration time to record an optimal Raman signal. Off-line references of total protein, *ds*DNA, total particles and infectious particles were aligned with their corresponding spectral measurements using matching timestamps. Separate PLS calibration models were developed for each parameter using the training set and evaluated through k-fold cross-validation (Table 1).

In the UF stage, the total protein model showed a high *R*^2^*Y* of 0.98 and a *Q*^2^*Y* of 0.91, reflecting good predictive capacity. This model used five latent variables, ranging from 1.72 to 3.12 mg/mL, with an *RMSEC* and *RMSECV* of 0.07 and 0.10, respectively. The total particle model achieved an *R*^2^*Y* of 0.96 and a *Q*^2^*Y* of 0.86 using four latent variables, ranging from 1.63 × 10^9^ to 1.44 × 10^10^ TP/mL. The *RMSEC* was 7.04 × 10^8^, and the *RMSECV* was 1.03 × 10^9^, demonstrating solid cross-validation performance. The infectious particles model was developed with four latent variables, ranging from 7.49 × 10^6^ to 1.21 × 10^10^ TU/mL, with an *RMSEC* of 1.59 × 10^9^ and an *RMSECV* of 2.13 × 10^9^. An *R*^2^*Y* of 0.78 combined with a *Q*^2^*Y* of 0.56 shows an unsuccessful correlation between the functional titer and the Raman spectra. The virus’s fragility makes the infectious titer a hard parameter to directly relate functional behaviour to structural changes and Raman signatures. The *ds*DNA model indicated an *R*^2^*Y* of 0.98 and a *Q*^2^*Y* of 0.86 using six latent variables. It ranged from 0.12 to 1.11 µg/mL, showing an *RMSEC* and *RMSECV* of 0.09. In the DF stage, the total protein model achieved an *R*^2^*Y* of 0.96 and a *Q*^2^*Y* of 0.85, employing four latent variables. Values ranged from 0.00 to 1.41 mg/mL, with an *RMSEC* of 0.09 and an *RMSECV* of 0.17. The *ds*DNA model recorded an *R*^2^*Y* of 0.96 and a *Q*^2^*Y* of 0.90 using four latent variables ranging from 0.00 to 0.20 µg/mL. The *RMSEC* was 0.01, and the *RMSECV* was 0.02, indicating good performance. No overfitting was detected in the models. In summary, the PLS models exhibited reliable performance across both UF and DF phases, confirming their efficiency in quantifying LV CQAs and total protein in this study.

#### 3.2.3. Progression of Buffer Exchange

**Figure 4:**
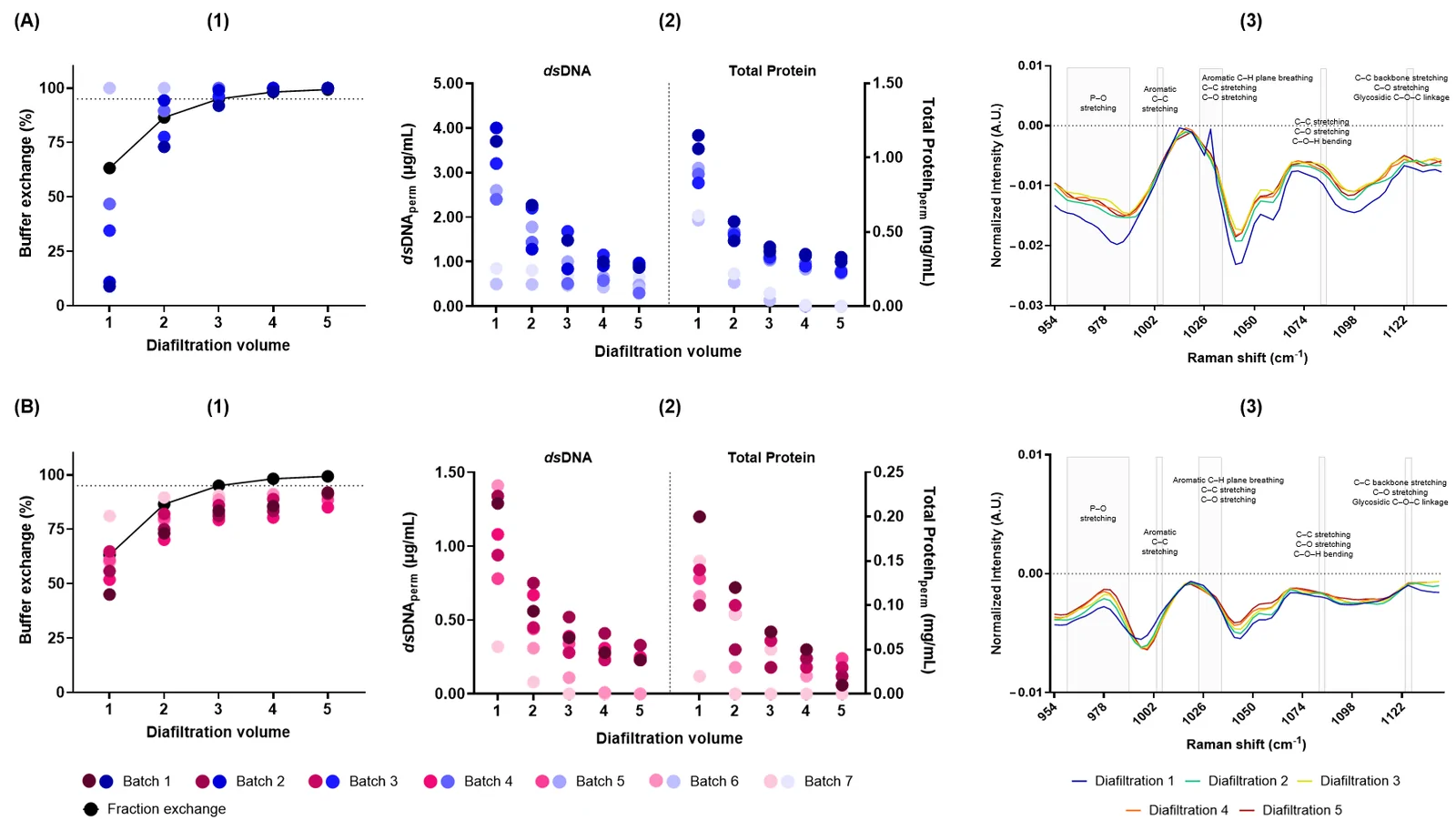
Evaluation of buffer exchange procedure through off-line and in-line measurements using concentrated and clarified batches of (**A**) AAV8 and (**B**) LV. (**1**) Percentage buffer exchange to PBS based on conductivity measurements of permeate with an ideal exchange rate of 95% (in dashed lines). (**2**) Concentration of removed impurities (*ds*DNA and free protein) and corresponding (**3**) Raman spectra of retentate mixture between 954 cm^−1^ and 1200 cm^−1^ at each diafiltration volume.

Incorporating a sensor in DF can provide further information about the extension of process time. The established procedures followed at-line measurement of conductivity, assuming an ideal buffer exchange of 95% at the third diafiltration volume. Standard procedures were compared with in-line Raman measurements to assess the abundance of chemical bonds associated with the change of spent medium to PBS (Figure 4).

The conductivity measurements of the permeate demonstrated a successful buffer exchange of 95% to PBS at the third diafiltration volume for all AAV batches (Figure 4(A1)). However, more diafiltration cycles are required to achieve a 95% buffer exchange in LV batches, and from the third diafiltration volume, there was inconsistent progression (Figure 4(B1)). Related to LV sensitivity, a trade-off between purity, percentage buffer exchange and diafiltration volumes is essential. For both viral vectors, a high removal of impurities (*ds*DNA and free protein) was reached at the third diafiltration volume (Figure 4(A2,B2)), despite the target number being mostly defined at the purity level. In LV batches, the impurity concentration progressively decreased along the diafiltration volumes. The low total protein concentration detected in the permeate indicated retention of the viral vectors, followed by titer confirmation. Looking into the spectral region, the P-O stretching of phosphate mineral between 960 cm^−1^ and 990 cm^−1^ stands from the C-C stretching of the aromatic ring of the phenylalanine at 1003 cm^−1^, the C-C stretching and the C-H breathing of the aromatic ring of the phenylalanine between 1025 cm^−1^ and 1030 cm^−1^, the C-O stretching and C-C stretching of glucose at 1085 cm^−1^, and C-C backbone stretching and C-O stretching of glucose at 1125 cm^−1^, considered a suitable region to evaluate the percentual buffer exchange. Surprisingly, only for AAV, the region between 1025 cm^−1^ and 1030 cm^−1^ shows a transition from spent medium to PBS buffer by the decrease in intensity of Raman shifts associated with the aromatic ring of phenylalanine. The phosphate bonds between 960 cm^−1^ and 990 cm^−1^ are more suitable for measuring the progression of buffer exchange in both viral vectors. The difference in amplitude was more significant from the first diafiltrate volume to the following volumes for AAV. It demonstrated a high percentage exchange with a decrease in phosphate bonds, attributed to a higher amount of phosphate molecules present in the medium than in PBS buffer. A progressive decrease in the vibration energy of phosphate bonds was observed in LV batches over the diafiltration volumes, indicating a progressive buffer exchange aligned with the removal of impurities.

### 3.3. Real-Time Monitoring of Ultra- and Diafiltration

The PLS models developed were used to monitor in real-time the UF/DF operation of one independent run for both viral vectors within the same operational and design space. Predictive values were tracked within and outside 95% confidence intervals to evaluate the Raman monitoring platforms. The model’s prediction capacity was assessed using *RPD* metrics, while *RE* was determined as a measure of magnitude between predicted values and off-line references. Besides the associated *RMSEC*, the models followed the operational trend stated by off-line references. In Supplementary Figure S1, the performance of each chemometric model is compared between predicted and measured values throughout UF/DF for both viral vectors.

**Figure 5:**
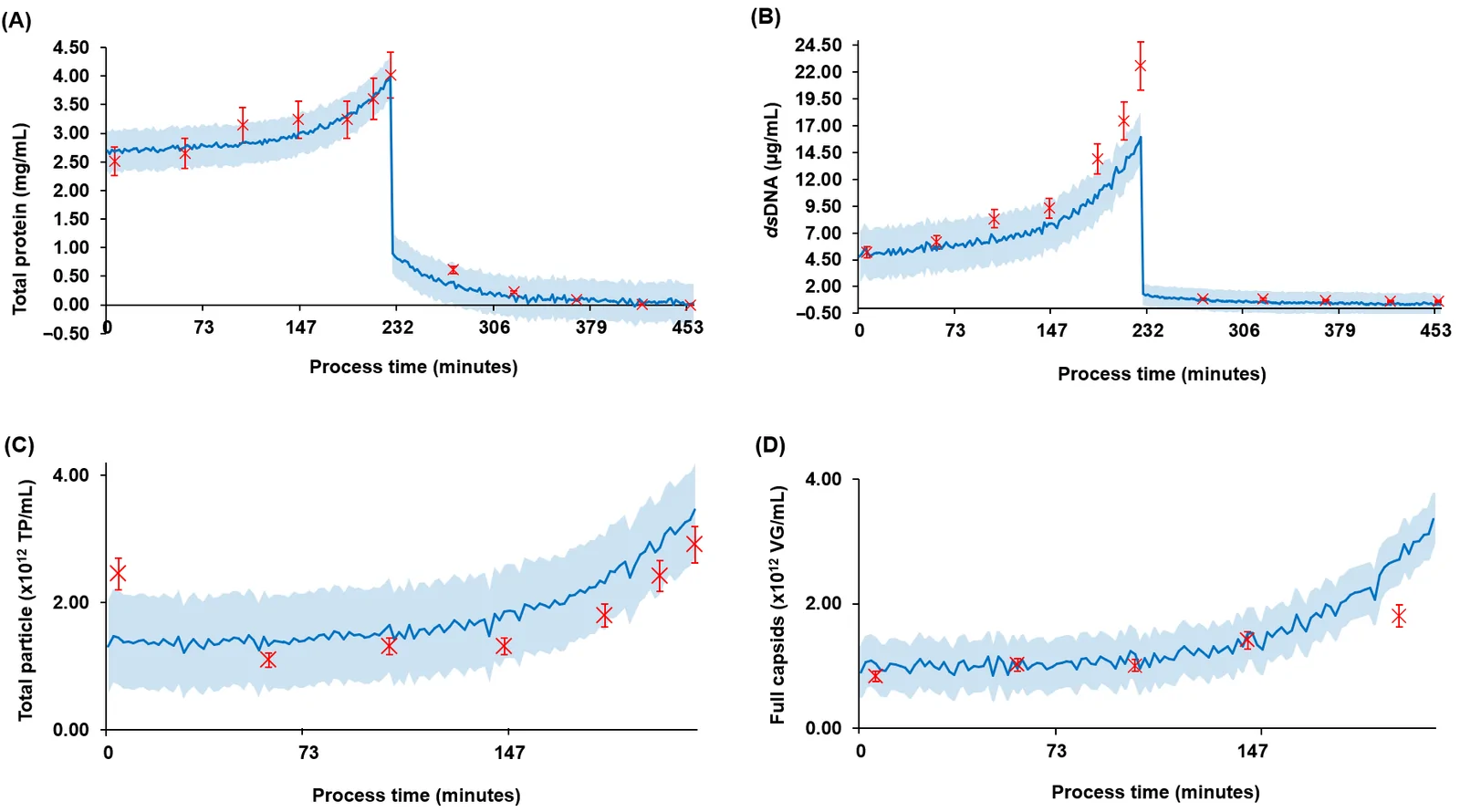
Real-time monitoring of UF/DF of AAV8 using Raman spectroscopy. Raman in-line predictions from PLS models of (**A**) total protein (mg/mL), (**B**) *ds*DNA (µg/mL), (**C**) total particle (TP/mL) and (**D**) full capsids (VG/mL) are represented as continuous lines (in dark blue) within *RMSEC* 95% confidence interval (in light blue) and off-line references as crosses (in red) within standard deviation. The UF operation finished after 217 min, followed by the DF operation until 453 min. The Raman monitoring platform was developed with seven UF/DF batches and validated against one independent batch.

The real-time monitoring of AAV8 revealed critical insights when comparing the continuous Raman monitoring curves with the off-line references (Figure 5). Following the normal UF tendency, the total protein concentration increased until 5.50 mg/mL at 232 min of operation (Figure 5A). An *RE* of 3.00% and an *RPD* of 5.20 indicated the model’s accurate prediction capacity and showed that PLS models support data variability (Table 1). In *ds*DNA monitoring, a sharp increase in concentration was detected (Figure 5B), resulting in an *RE* of 16.0% and an *RPD* of 1.90. The total particle and full capsid concentrations demonstrated a strong correlation with Raman monitoring for capturing the UF trend steadily (Figure 5C and Figure 5D, respectively). In the final operation phase, the total particle titer reached values of approximately 2.90 × 10^12^ TP/mL, with an *RE* of 16.0% and an *RPD* of 1.60. The model for AAV8 total particle titer performed fairly. The incorporation of more batches into the training set would improve the capture of process variability and, ultimately, lead to more robust models. The full capsid titer stabilised around 4.00 × 10^12^ VG/mL, with an *RE* of 10.0% and an *RPD* of 2.10. As a result, the model for AAV8 full capsid performed accurate predictions and accommodated process variability. Outliers were identified in the off-line references, and those values were not included. By exposing the operation trend, underestimated samples were easily detected in off-line references due to sampling or quantification errors. During the DF phase, the permeability of impurities such as free proteins and *ds*DNA was screened. An *RE* of 27.0% and an *RPD* of 0.40 show a poor prediction capacity in the model for *ds*DNA. The model must be improved with the incorporation of more batches in the training set. However, the low *ds*DNA content (close to the limit of quantification of the standard analytical method) combined with the intrinsic fluorescence of AAV8 hinders accurate *ds*DNA quantification and, thus, limits Raman predictions. Visual inspection after DF proved a complete buffer exchange and the intrinsic fluorescence of AAV8. On the contrary, the model for total protein provided an *RE* of 17.0% and an *RPD* of 2.50, showcasing its accurate prediction capacity. The Raman monitoring platform herein developed showed the efficiency of Raman spectroscopy to monitor in real-time UF/DF of AAV8, accounting for outlier identification.

**Figure 6:**
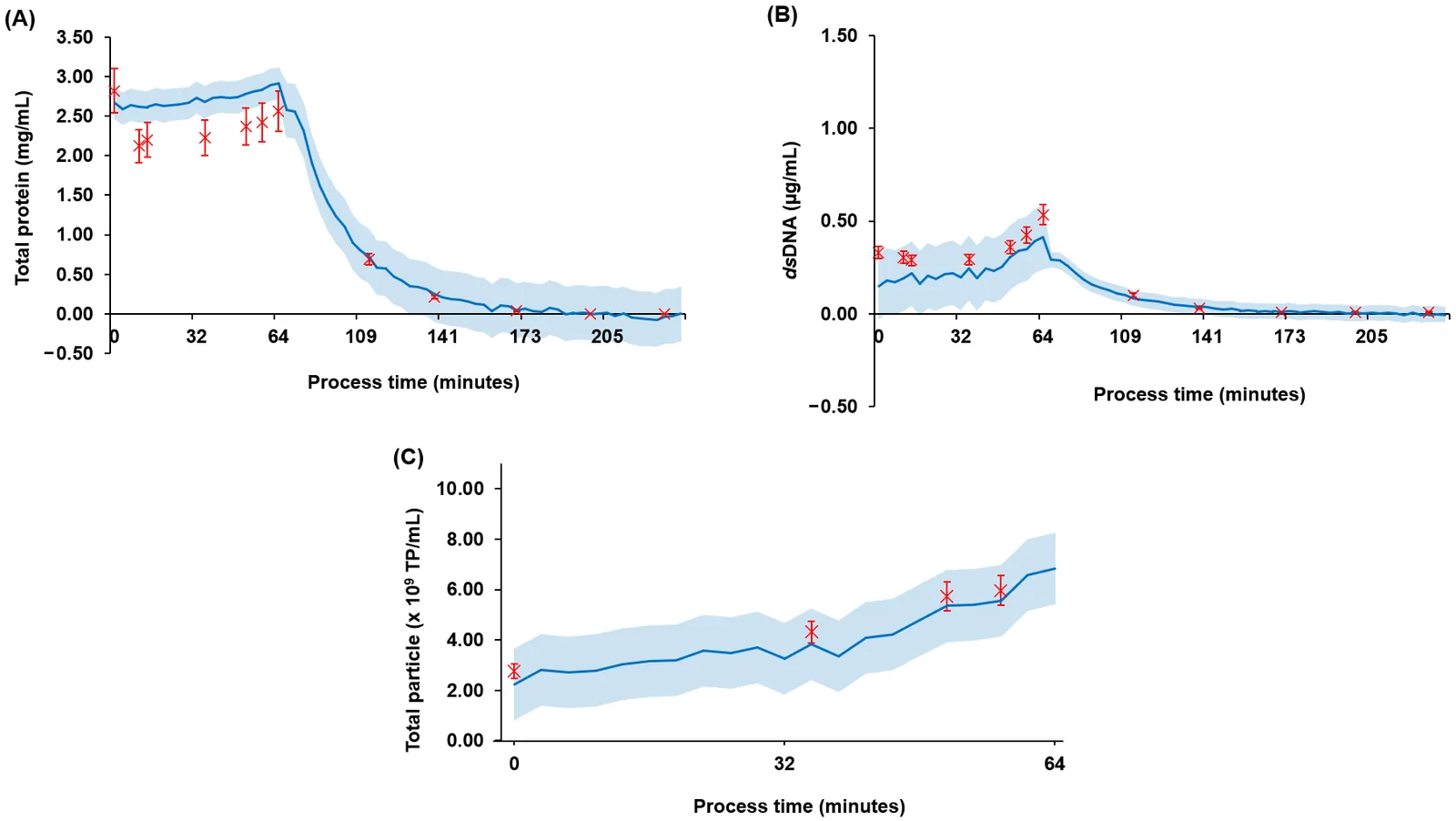
Real-time monitoring of UF/DF of LV using Raman spectroscopy. Raman in-line predictions from PLS models of (**A**) total protein (mg/mL), (**B**) *ds*DNA (µg/mL) and (**C**) total particle (TP/mL) are represented as continuous lines (in dark blue) within *RMSEC* 95% confidence intervals (in light blue) and off-line references as crosses (in red) within standard deviations. The UF operation finished in the first 65 min, and the DF operation proceeded until 240 min. The Raman monitoring platform was established with seven UF/DF batches and assessed with one independent batch.

When evaluating the Raman monitoring platform for LV, the operating trend was effectively captured, in spite of some observed fluctuations in off-line references (Figure 6). At the end of UF, the total protein concentration reached 2.60 mg/mL, with an *RE* of 13.0% and an *RPD* of 0.61 (Figure 6A and Table 1). The PLS model for total protein showed poor predictive performance, requiring further improvement. More batches need to be included to properly capture process variability derived from production. The quantification of *ds*DNA varied from 0.30 to 0.50 µg/mL (Figure 6B), with a high *RMSEC* compared with the DF model. The combination of the Raman-active property of LV with medium fluorescence may compromise Raman predictions by smoothing minority Raman shifts. This can slightly induce a systematic error in data collection and lead to an *RE* of 18.0% and an *RPD* of 1.26. The model for *ds*DNA showed a poor predictive capacity; it must be improved for monitoring purposes. In UF, the total particle concentration steadily increased until it reached approximately 6.00 × 10^10^ TP/mL (Figure 6C). An *RE* of 7.00% and an *RPD* of 1.60 showed the model’s fair prediction capacity. Further improvement may be considered, in spite of some off-line references that were identified as outliers. These results indicate the efficiency of Raman spectroscopy in monitoring viral particle titer. In contrast, free protein and *ds*DNA were minimised during DF, where the predicted values closely followed off-line references. For free protein, an *RE* of 4.00% and an *RPD* of 10.20 demonstrated the model’s accurate prediction capacity. The *ds*DNA clearance was effectively monitored in the DF phase with an *RE* of 1.00% and an *RPD* of 4.00. This highlights the model’s accurate prediction capacity in monitoring the *ds*DNA concentration in a buffer with low fluorescence compared with spent media. The visual inspection detected a complete buffer exchange after DF and no significant intrinsic fluorescence in LV. The Raman monitoring platform for LV proved to monitor UF/DF operation in real-time within a model space designed to accommodate process variability.

## 4. Discussion

Each molecule has a characteristic Raman spectrum with a unique pattern of vibrational modes that identifies its composition and structure [22]. The initial spectral characterisation of each viral vector exploited in this work demonstrated that Raman spectroscopy is sensitive to viral vectors, and AAV8 is more Raman-active than LV. This could be associated with their protein composition. The successful characterisation of each CQA was performed in the protein fingerprint area. The dominant amide vibrational signature in AAV8 Raman spectra, compared with LV Raman spectra, reflects the composition of the AAV8 protein capsid, composed of three viral proteins (VP1, VP2 and VP3) in an icosahedral configuration [50]. The intensified Raman shifts in the AAV8 spectra suggest a higher surface protein density and content of aromatic amino acids within AAV8 than LV. When analysing the AAV8 capsid, approximately 20% of the structure has aromatic amino acids (AAV8 capsid Uniprot ID Q8JQF8). The Raman shifts between 1238 cm^−1^ and 1253 cm^−1^ may be highlighting the exposure of VP side-chain amino acids within β-sheets barrel motifs characteristic of AAV8. This contributes to increased Rayleigh scattering and fluorescence background [51], resulting in a baseline shift. Contrary to AAV8, LV has a lipidic envelope with embedded receptors and transmembrane proteins derived from the host cell, together with the VSV-G protein, which is the element responsible for virus transduction [52,53]. However, protein structural analysis in LV is more complex since host cell-surface proteins are host-dependent and predominant in the virus envelope relative to the envelope protein. Considering HIV-1 virus as an example, the host-cell proteins found in virions are more abundant than viral proteins and come from a broad range of classes [53,54]. However, LV shows low intrinsic fluorescence when compared with AAV8, likely associated with protein interactions on the viral vector surface.

After characterisation of the viral spectra and evaluation of Raman sensitivity to measure the viral vectors’ CQA, the technology was applied in a downstream unit operation. UF/DF is a well-established technique in the downstream process of viruses and viral vectors for gene therapy. It enables viral retention and impurity clearance without detection of viral particles in the permeate [55]. Coupling the technique with a robust monitoring platform allows operation according to specifications, avoiding under- and over-processing. Ultimately, it contributes to product quality by reducing the probability of aggregation relative to extended concentration, unnecessary DF cycles and excessive shear. In this work, for both viral vectors, three diafiltration volumes are considered efficient to formulate viral vectors in PBS buffer (unlike AAV, which reaches 95% buffer exchange, over-processing influences LV functional titer without further reduction in impurity removal). Analysis of the Raman spectra aligned with standard at-line measurements of conductivity and impurity removal. This strategy can be an extra parameter to follow UF/DF operation and can be further used to adjust the operation to the requirements. In this specific case, the reduction in diafiltration volumes reduces buffer consumption and associated costs. A direct “visualisation” of the process by a real-time monitoring platform helps identify outliers in the off-line reference dataset that could lead to misinterpretations of UF/DF operation. This reinforces the importance of using multiple analytical methods to characterise complex biopharmaceuticals. Combining multiple parameter readings into a single software platform makes Raman spectroscopy a powerful PAT tool. Nevertheless, a successful application is dependent on the fluorescence of the mixture, and an adjustment in the integration time may be required. To operate the Raman probe inside the optimal dynamic range, the integration time for AAV8 was reduced to 8 s, and for LV, it was maintained at 35 s. Graf et al. (2022) [56] and Rolinger et al. (2023) [41] reported an integration time lower than 20 s to measure in-line mAbs according to study specifications. Looking at the *RE* of proteins and *ds*DNA during DF, the error below 5% for LV contrasted with the substantially higher error for AAV, ranging from 17 to 27%, at comparable concentrations. Differences in Raman integration time become particularly critical when operating near the limit of detection of Raman spectroscopy, where a low integration time yields a superior signal-to-noise ratio. Longer integration times are essential for detecting molecules in traces, maintaining analytical precision and minimising measurement uncertainty. Comparing the prediction capacity of the LV models for *ds*DNA between UF and DF showed the influence of medium/buffer fluorescence on Raman’s sensitivity to quantify low concentrations of CQAs. Overall, the *ds*DNA PLS model seems to be the most affected by fluorescence and shows how fluorescence plays a critical role in monitoring processes using Raman spectroscopy. Therefore, applying Raman spectroscopy to monitor other downstream process units, where the inherent fluorescence of buffers is reduced (e.g., in chromatography), would be beneficial for further improving process understanding.

When establishing a monitoring platform, a large dataset that captures all the sources of variability in the process is required. For trend-monitoring purposes, an *RE* lower than 20% is acceptable to identify relative changes and deviations in operation, whilst for process control, an *RE* lower than 10% is required to ensure accurate predictions. The model prediction capacity can be improved by (1) incorporating more batches into the dataset, (2) detailed experimental design and (3) development time. In this work, the PLS models developed are a proof-of-concept based on a short dataset of 1050 Raman spectra with almost 50 samples characterised off-line for each process stage. Effective monitoring platforms for UF/DF of viral vectors showed accurate models for some parameters. The variability identified in PCA analysis was derived from biological replicates (three for AAV8 and five for LV) and changes in UF/DF cassette over-runs. As a consequence, the biological variability induced exit of the batch from the model space due to the influence of unusual production yields and product quality, and the change in cassette promoted slight parallel shifts along batches in the model space related to membrane properties, age and packaging. To mitigate model underperformance, a dataset with 20 to 30 batches would properly capture the batch-to-batch variability of the process, while pre-processing methods help to adapt the model to data variability. Despite the biological variability being majority-controlled in upstream processing, it may result in unexpected impacts on downstream processes. Even though cross-validation analysis indicates no overfitting within the model space, the pre-processing methods used impact PLS model performance. The choice of normalisation methods, Savitzky–Golay filter parameters, order of pre-processing and selection of the spectral range are some pre-processing strategies exploited during the model-building phase when developing outperforming models. According to Bocklitz et al. (2011) [57] and Rinnan et al. (2009) [58], other pre-processing combinations could be investigated to reduce baseline fluorescence. Increasing the training set, in combination with further pre-processing optimisations and potential refinement of integration time, could contribute to enhancing the robustness and accuracy of underperforming PLS models.

The implementation of PAT tools in the biopharmaceutical industry aims to control the bioprocesses of highly variable systems for high-value-added products in accordance with CQA target requirements for improving product quality. Consistency across scales and batches indicates operational robustness and reduces the propensity for batch rejection and investment losses. Short production cycles with high performance and recorded information foster reductions in costs for advanced therapies. It is strongly encouraged to establish PAT tools in biomanufacturing during the process development phase. However, a dataset with 30 to 50 batches is required to successfully establish a robust and accurate monitoring platform for industrial application. From successful cases reported in the literature, Raman spectroscopy is adaptable to the processing stage and to different products, monitoring multiple CQAs simultaneously. The use of PAT in downstream processes will support the change from batch to continuous manufacturing in gene and oncolytic therapy and, ultimately, will be extended to vaccine development. The transferability of these platforms across facilities remains one of the current challenges in the field. Schini et al. (2026) [59] developed an automated method using the dynamic orthogonal projection algorithm to monitor the production of mAbs in fed-batch with pre-existing models for batch production. The transfer learning successfully adapted the PLS models to a new process, even with baseline shifts and Raman spectral variations derived from feed additions. Additionally, a control system based on the Raman signal was established to automatically add glucose when its concentration decreases below 5.5 g/L.

In this work, batch-to-batch variability was identified as the principal source of spectral variation and explained the model space, while adapting pre-processing strategies seems to be an appropriate approach to increase model performance. The increase in the design space in which the model operates allows to attenuate the influence of batch-to-batch variability on the model prediction capacity and amplified the potential use of Raman spectroscopy in bioprocessing engineering. A real-time monitoring tool acknowledges the operation trend and easily identifies outliers in an off-line reference dataset. When operating outside the model space, it identifies unmatched requirements of biological material in early stage and reinforces the need to adjust the UF and/or DF unit of operation towards the target in an optimised process. In the case of UF, it contributes to understanding the demand for reducing or extending the operation time to reach the expected titer, the occurrence of cassette clogging due to the reduction in viral particle titer in retentate, and the reason for unexpected exponential increases in feed and TMP pressure. Meanwhile, in the case of DF, it allows matching the desired *ds*DNA clearance to the number of diafiltration volumes, assessing buffer consumption and associated costs. Moreover, the Raman monitoring platform directly provides insight into DF operation design by indicating retention of impurities resulting from improper membrane cut-off. Particularly in this study, the membrane cut-offs chosen were suitable for UF/DF operation of AAV8 and LV since impurities from clarified material were not retained, pressure increases were not detected, and the LV functional titer followed the UF trend, remaining infectious throughout the UF/DF operation. With all due consideration, Raman spectroscopy represents a suitable PAT tool for real-time monitoring of UF/DF of viral vectors with potential application in other downstream operations units.

## 5. Conclusions

This study successfully showed the capacity of Raman spectroscopy to distinguish viral vector spectra by capturing structural differences and its application for developing a real-time monitoring platform for UF/DF of AAV8 and LV. The modelling approach proved to be a suitable strategy to establish chemometric models for monitoring AAV and LV processes. The correlation of Raman spectra data with off-line references underscores the potential of these monitoring platforms to enhance process efficiency and product quality.

The integration of real-time monitoring capabilities into bioprocessing of viral vectors reflects a significant advance towards autonomous manufacturing in the biopharmaceutical sector. By enabling continuous feedback on CQAs, Raman spectroscopy contributes to the robustness of filtration processes by providing valuable insight into batch-to-batch variability, process knowledge and timely decision making. Future work will focus on improving models’ accuracy through expansion of dataset, optimisation of acquisition settings and exploration of other pre-processing methodologies.

## Figures and Tables

**Table 1:** Statistical analysis of PLS models’ performance in monitoring UF/DF of AAV8 and LV after calibration with the training set and after validation with an independent UF/DF batch of AAV8 and LV. The parameters measured during the operation were total particles (TP/mL), full capsids (VG/mL), infectious particles (TU/mL), *ds*DNA (µg/mL) and total protein (mg/mL). *N*: number of reference samples; IC_95%_: confidence interval at 95%; *R*^2^*Y*: cumulative explained variance; *Q*^2^*Y*: cumulative predicted variance; *RMSEC*: root-mean-square error of calibration; *RMSECV*: root-mean-square error of cross-validation; *RMSEP*: root-mean-square error of prediction; *RPD*: ratio of prediction to deviation; and *RE*: relative error.

			Model Calibration								Model Validation				
Viral Vector	Step	Parameter	*N*	Latent Variables	Min. Value	Max. Value	*R* ^2^ *Y*	*Q* ^2^ *Y*	*RMSEC*	*RMSECV*	*N*	IC_95%_	*RMSEP*	*RPD*	*RE* (%)
AAV8	UF	Total Particle	26	4	2.51 × 10^11^	4.09 × 10^12^	0.93	0.82	3.68 × 10^11^	5.60 × 10^11^	5	[0.48, 4.19] × 10^12^	4.80 × 10^11^	1.60	16.0
		Full Capsids	33	5	1.35 × 10^11^	4.89 × 10^12^	0.98	0.90	1.97 × 10^11^	4.70 × 10^11^	4	[0.45, 3.76] × 10^12^	1.40 × 10^11^	2.10	10.0
		Total Protein	36	4	1.90	5.69	0.99	0.97	0.12	0.17	6	[2.30, 4.32]	0.10	5.20	3.00
		*ds*DNA	36	4	3.57	30.39	0.98	0.94	1.19	2.05	6	[0.29, 18.34]	3.70	1.90	16.0
	DF	Total Protein	35	5	0.00	1.15	0.94	0.67	0.08	0.17	5	[−0.37, 1.18]	0.10	2.50	17.0
		*ds*DNA	34	3	0.30	4.00	0.87	0.75	0.37	0.51	5	[−0.72, 2.25]	0.20	0.40	27.0
LV	UF	Total Particle	32	4	1.63 × 10^9^	1.44 × 10^10^	0.96	0.86	7.04 × 10^8^	1.03 × 10^9^	4	[1.30, 8.25] × 10^9^	4.30 × 10^8^	1.60	7.0
		Infectious Particles	32	4	7.49 × 10^6^	1.21 × 10^10^	0.78	0.56	1.59 × 10^9^	2.13 × 10^9^	4	[2.00, 10.20] × 10^9^	2.38 × 10^9^	1.36	20.0
		Total Protein	35	5	1.72	3.12	0.98	0.91	0.07	0.10	6	[2.39, 3.09]	0.38	0.61	13.0
		*ds*DNA	34	6	0.12	1.11	0.98	0.86	0.09	0.09	6	[−0.02, 0.40]	0.08	1.26	18.0
	DF	Total Protein	33	4	0.00	1.41	0.96	0.85	0.09	0.17	5	[−0.42, 3.11]	0.03	10.20	4.00
		*ds*DNA	34	4	0.00	0.20	0.96	0.90	0.01	0.02	5	[−0.05, 0.33]	0.01	4.00	1.00

## Data Availability

The data supporting the findings of this study are available within this article and the Supplementary Material. The reagents used in this study (e.g., cell line, plasmids and viral vectors) are only readily available internally to the authors’ institutions’ staff for R&D purposes.

## Supplementary Materials

The following supporting information can be downloaded at https://www.mdpi.com/article/10.3390/pharmaceutics18081016/s1. Figure S1: Performance analysis of the monitoring platforms for UF/DF of (**A**) AAV8 and (**B**) LV. Representative comparison between off-line references and predicted values for viral vector titer, total protein and *ds*DNA in calibration (n = 7) and validation (n = 1) sets through (**1**) parity plots with an accepted 20% deviation (dashed line zone), (**2**) residual plots and (**3**) time-resolved prediction traces.

## Abbreviations

The following abbreviations are used in this manuscript:

APIs	Active Pharmaceutical Ingredients
AAV	Adeno-Associated Virus
sbAb	Bispecific Monoclonal Antibody
CPPs	Critical Process Parameters
CQAs	Critical Quality Attributes
DF	Diafiltration
EMA	European Medicines Agency
FDA	Food and Drug Administration
GFP	Green Fluorescent Protein
LV	Lentiviral Vector
mAbs	Monoclonal Antibodies
PLS	Partial Least Squares
PBS	Phosphate-Buffered Saline
PCs	Principal Components
PCA	Principal Component Analysis
PAT	Process Analytical Technology
*Q*^2^*Y*	Cumulative Predicted Variance
QbD	Quality by Design
*R*^2^*Y*	Cumulative Explained Variance
*RPD*	Ratio of Prediction to Deviation
*RE*	Relative Error
RMSEC	Root-Mean-Square Error of Calibration
RMSECV	Root-Mean-Square Error of Cross-Validation
RMSEP	Root-Mean-Square Error of Prediction
SNV	Standard Normal Variate
TFF	Tangential Flow Filtration
TP	Total Particle
TMP	Transmembrane Pressure
UF	Ultrafiltration
VP	Viral Protein
