# Supplementary material for: Applying Raman Spectroscopy for Real-Time Monitoring of Ultra- and Diafiltration Steps in Viral Vector Purification
Source: Pharmaceutics. 2026 Aug 17;18(8):1016. doi: 10.3390/pharmaceutics18081016 (PMC13516993; doi:10.3390/pharmaceutics18081016)
Supplement: Supplementary file 1 [file pharmaceutics-18-01016-s001.zip › pharmaceutics-4396675-supplementary.pdf]

pharmaceutics

Supplementary material

Applying Raman Spectroscopy for Real-Time Monitoring of Ultra- and Diafiltration Steps in Viral Vector Purification

S1. Supplementary material: Analysis of PLS model performance

Complementary analysis of the monitoring platform's capacity to predict viral vector titer (total titer, full capsids for AAV8 and functional titer for LV), total protein and *ds*DNA values, through the comparison between observed values (off-line reference) and predicted values in calibration and validation sets. The principle of parity plots shows the alignment between predicted values and observed values, whilst the residual plots demonstrate the residual random distribution. These identify if the model adequately captures the underlying data structure. The time-resolved prediction traces highlight how a model accurately reproduces the temporal dynamics of a system, showing responses and behaviours from off-line references and predicted values.

(A)

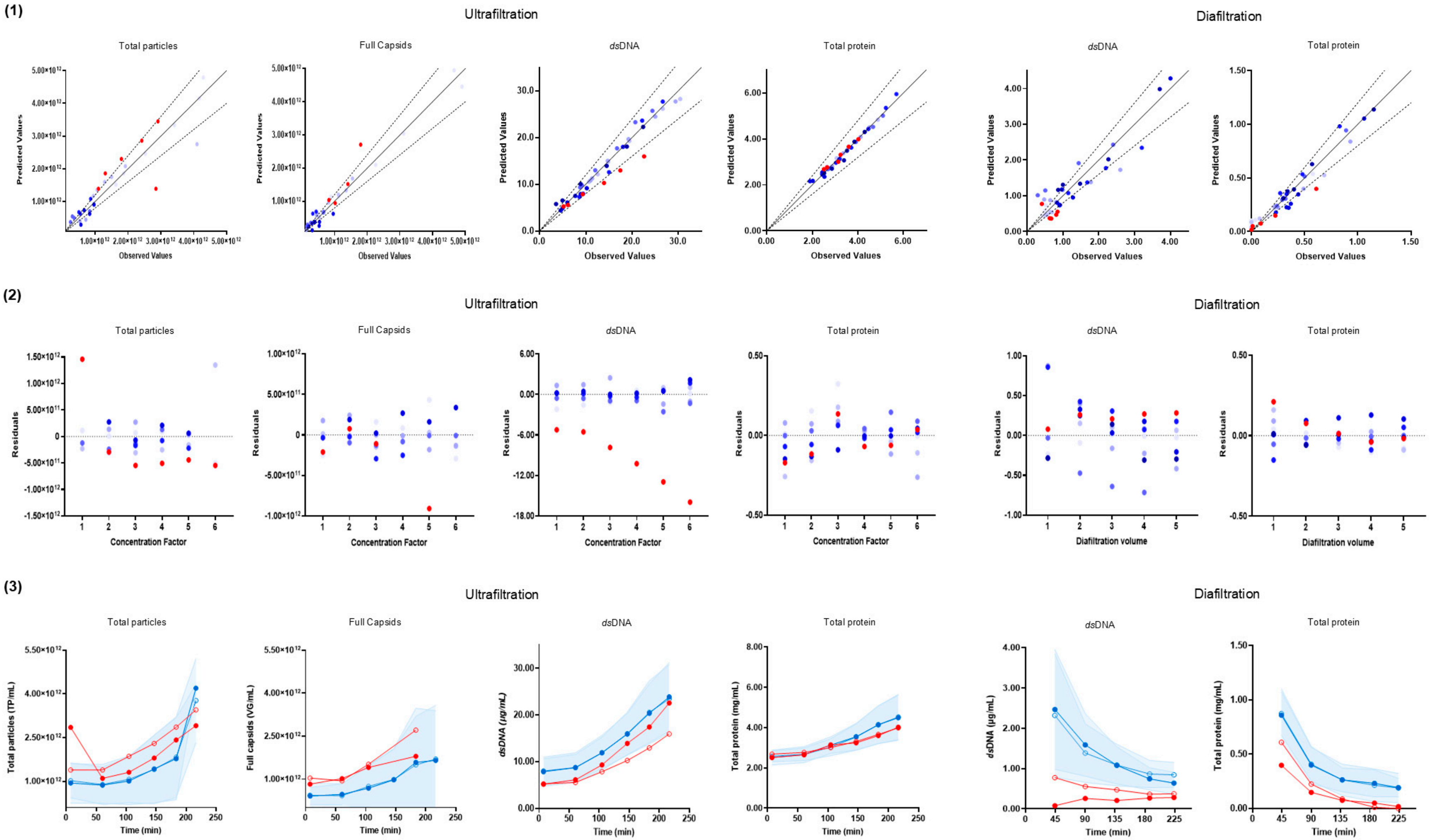

(1)
Ultrafiltration
Diafiltration
Total particles
Full Capsids
dsDNA
Total protein
dsDNA
Total protein
Predicted Values
Observed Values
(2)
Ultrafiltration
Diafiltration
Total particles
Full Capsids
dsDNA
Total protein
dsDNA
Total protein
Residuals
Concentration Factor
Diafiltration volume
(3)
Ultrafiltration
Diafiltration
Total particles
Full Capsids
dsDNA
Total protein
dsDNA
Total protein
Total particles (TP/mL)
Full capsids (VG/mL)
dsDNA (µg/mL)
Total protein (mg/mL)
dsDNA (µg/mL)
Total protein (mg/mL)
Time (min)

continuation *Supplementary Figure S1*

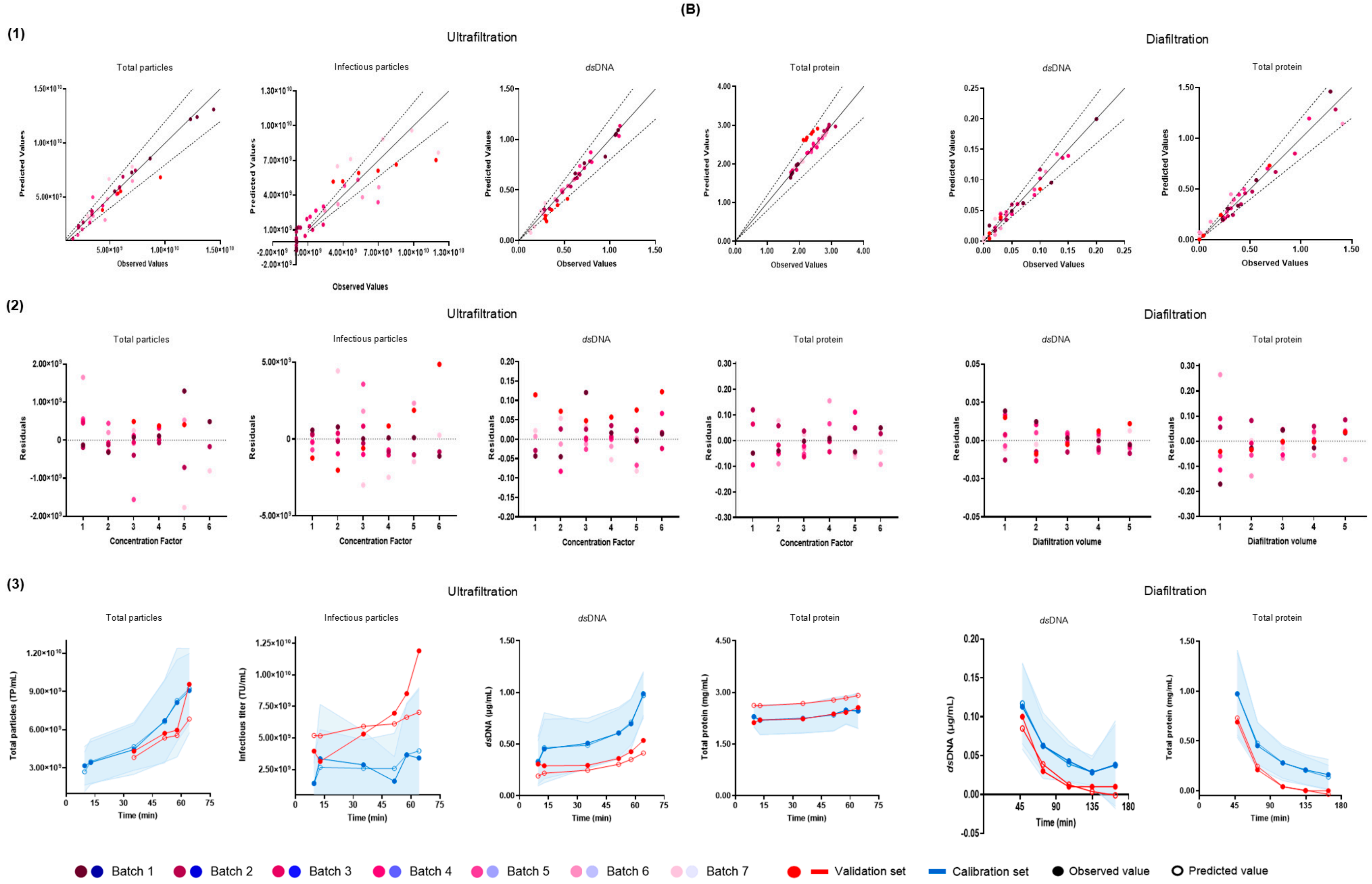

Supplementary Figure S1. Performance analysis of the monitoring platforms for UF/DF of (**A**) AAV8 and (**B**) LV. Representative comparison between off-line references and predicted values for viral vector titer, total protein and *ds*DNA in calibration (n = 7) and validation (n = 1) sets through (**1**) parity plots with an accepted 20 % deviation (dashed line zone), (**2**) residual plots and (**3**) time-resolved prediction traces.
